# Organic nitrogen steadily increasing in Norwegian rivers draining to the Skagerrak coast

**DOI:** 10.1038/s41598-020-75532-5

**Published:** 2020-10-28

**Authors:** A. Deininger, Ø. Kaste, H. Frigstad, K. Austnes

**Affiliations:** 1 Centre for Coastal Research, University of Agder, Kristiansand, Norway; 2 Norwegian Institute for Water Research (NIVA), Oslo, Norway

**Keywords:** Element cycles, Climate-change impacts, Environmental chemistry, Environmental impact, Limnology, Marine chemistry

## Abstract

Declining atmospheric nitrogen (N) deposition, through reduction in the direct input of inorganic N, may result in less inorganic N being leached from soils to freshwaters (dissolved inorganic N = DIN). Declining sulphur deposition, through reducing the ionic strength in soil water, increases the solubility and mobility of organic soil compounds and may result in increased leaching of organically bound N to freshwaters (total organic N = TON). It is unknown to which extent these two independents and opposing trends, i.e. DIN decline versus TON increase, may affect the nutrient balance (load, stoichiometry) of river water draining into coastal zones. By combining long-term atmospheric and riverine monitoring data of the five major Norwegian rivers draining to the Skagerrak coast, we show that over the past 27 years (1990–2017) river water nutrient composition, and specifically N stoichiometry has been steadily shifting from inorganic to organic fractions, with correlations to changes in human pressures (air pollution), but especially climate (precipitation, temperature, discharge). This shift in nutrient quality may have large consequences on the nutrient cycling in both freshwater and coastal ecosystems and illustrates the complex interactions of multiple stressors (here: N deposition, S deposition, and climate change) on aquatic ecosystems.

## Introduction

From fresh to salty, aquatic systems are strongly affected by human influence on air and land^1–3^. Emission reduction in central Europe has resulted in declining atmospheric nitrogen (N) deposition and consequent declining concentrations of inorganic N (dissolved inorganic N = DIN) in Scandinavian lakes and rivers^4–6^. In parallel, declining sulphur (S) deposition, due to measures to reduce the acidification of freshwaters, has increased the solubility and mobility of organic soil compounds, and resulted in an increased leakage of organically bound N from soils to aquatic ecosystems^7–9^ (total organic N = TON). How these two independent and opposing trends (DIN decline versus TON increase) may affect the nutrient balance (load, stoichiometry) of river water draining into coastal zones has to date not been systematically investigated^6^.

A darkening of coastal waters has been observed in the North Sea and Skagerrak over the past decades^10,11^. It is hypothesized that this phenomenon might be related to the increased riverine discharge of freshwater (i.e. reduced salinity), as well as the increased discharge of terrestrial organic matter into coastal zones^3,12^. As the leaching of organic material into aquatic systems is expected to continue due to the numerous interacting drivers affecting land–water interactions, such as climate change (e.g. increased precipitation, warming)^7,12,13^, and other human activities (e.g. land use changes, population growth)^3,13,14^, filling the knowledge gap as to whether nutrient loads, and nutrient stoichiometry have been systematically changing over the past decades is of high urgency to improve our understanding of multiple stressor effects on aquatic ecosystems.

In order to understand how alterations of biogeochemical cycles may affect ecosystems, e.g. via bottom-up effects on food webs, analysing the relative abundance of key elements such as carbon (C), N and phosphorus (P) has proven to be a powerful tool for ecologists (ecological stoichiometry)^15,16^. Traditionally, the total nutrient content has been used as a predictor for the nutrient and limitation status of aquatic systems (e.g. total N versus total P). However, not all total nutrient constituents may be equally bioavailable^17–19^. Instead, various fractions, such as inorganic N or organic N, may be more accurate proxies for determining the bioavailability of a given nutrient pool^17,18,20^. Further, especially regarding the total organic matter pool, a majority of studies has to date focused on the distribution, flux and bioavailability of C, and to a less extend on N, but also P^17,20^. However, given the recent observations that the various mobilized fractions of organic material in boreal ecosystems may be much more bioavailable than previously assumed^17,21^, and that terrestrial riverine input may strongly affect coastal zones^22–24^, it is timely to investigate the existence of systematic long-term shifts in inorganic to organic N ratios in boreal areas with previously high and now declining S and N deposition^22^.

Lastly, the long-term fate of the deposited N onto boreal catchments is still unresolved, as to date no strong leaching of inorganic N has been reported across Scandinavia^5^. However, given the increased runoff of organic material, part of the N stored in the terrestrial catchments may leach into aquatic systems in organic form^22,25,26^. Thus, while the increased leaching of organic N may largely relate to the increased runoff of organic material in general (i.e. induced by declining acidification, increasing precipitation), this leaching may be further enhanced if the terrestrial systems have been enriched in N (i.e. induced by N deposition)^26^. We suggest that the total organic carbon (TOC) :TON ratio may be used to indicate whether the terrestrial system has been enriched in N over time, where declining TOC:TON may reflect a decline in soil and litter C:N.

**Figure 1:**
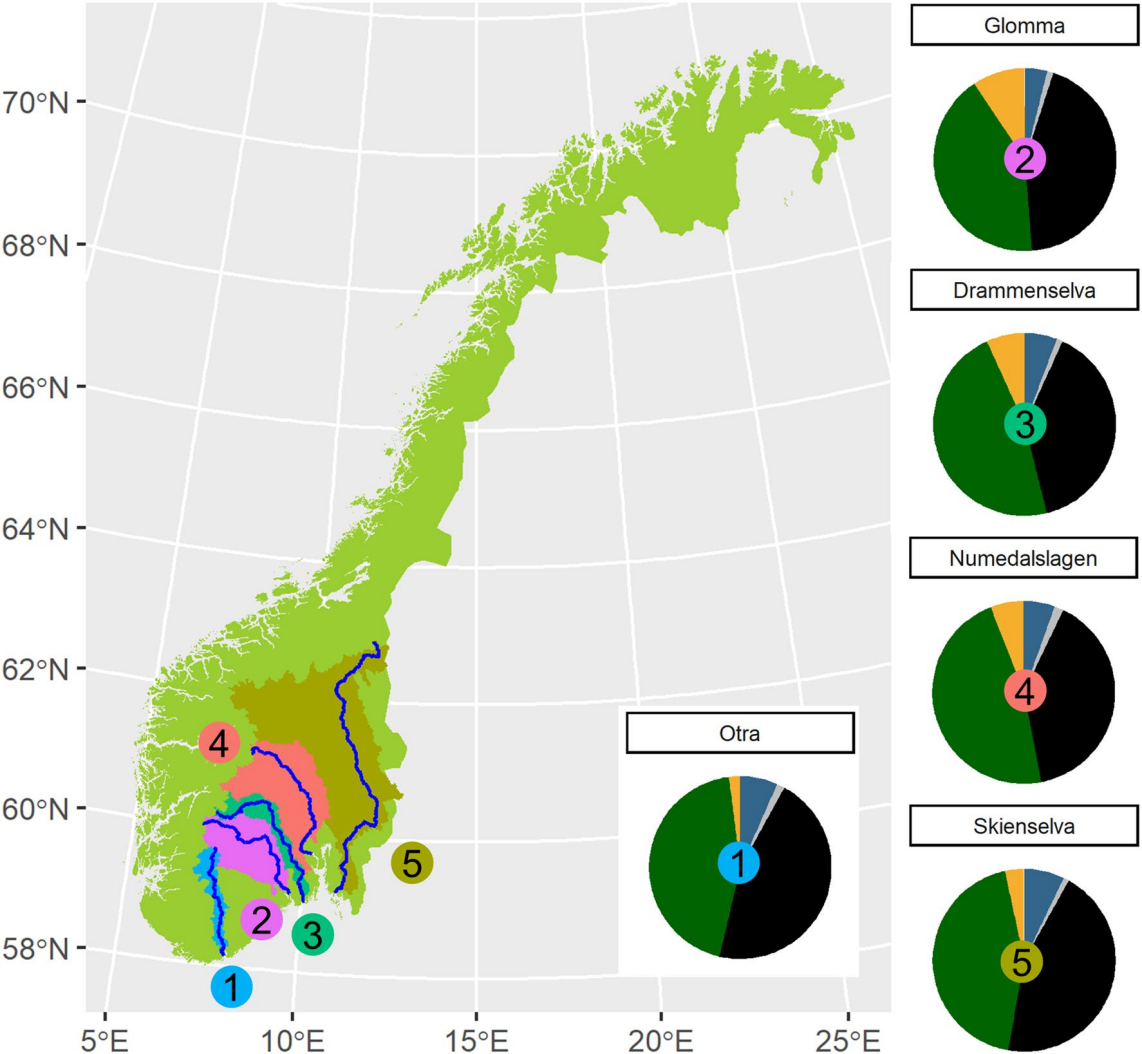
Map of Norway illustrating the location of the five monitored rivers draining to the Skagerrak coast, and their catchment characteristics (yellow: agriculture, green: forest, black: mountain, grey: urban, blue: water). The map was generated using the freely available software R (R CoreTeam 2020, Version 4.0.2, https://www.R-project.org/).

The aim of this study was to assess trends and drivers of riverine nutrient loads and nutrient stoichiometry in areas experiencing declining S and N deposition, as well as climate change (here: increased precipitation, temperature). Combining atmospheric and riverine long-term monitoring data (1990–2017) from the five major Norwegian rivers draining into the Skagerrak coast (Fig. 1) we hypothesize that:

Declines in N and S deposition have resulted in decreasing riverine loads of DIN and increasing loads of TON over the past decades.
(a) DIN:TON ratios have decreased in response to declining DIN and increasing TON loads over the past decades.
  (b) The decrease in DIN:TON has been stronger than the decrease in DIN:TP, and especially TN:TP over the past decades.
TOC:TON ratios have been declining in recent years as a result of increased N enrichment of terrestrial systems following decades of N deposition.

## Results and discussion

**Figure 2:**
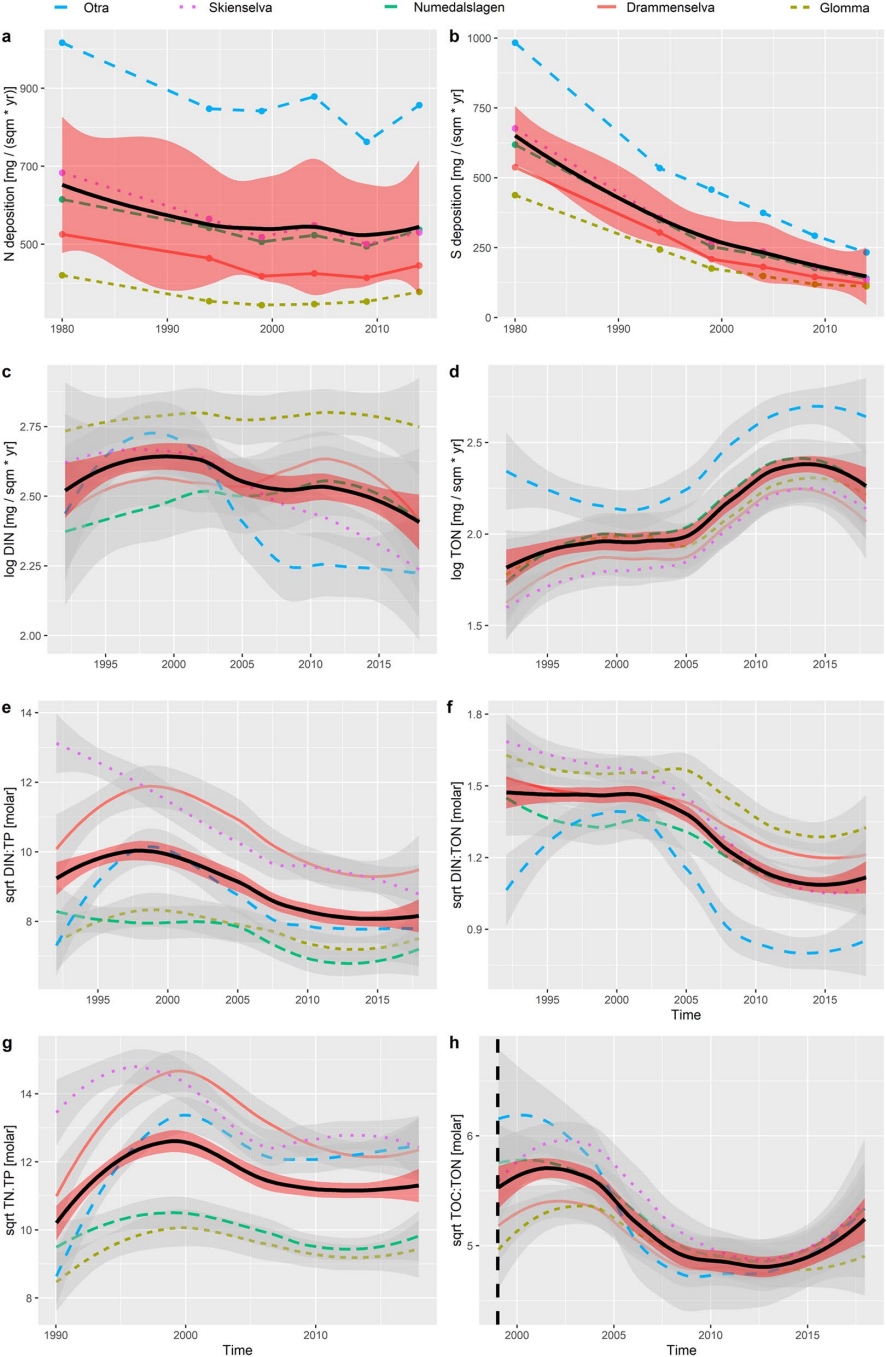
Time series of (**a**) nitrogen- and (**b**) sulphate deposition (both: 1980–2014), (**c**) dissolved inorganic nitrogen (DIN), (**d**) total organic nitrogen (TON), (**e**) DIN:TP ratio, (**f**) DIN:TON ratio, (**g**) TN:TP ratio (all: 1990–2017) and (**h**) total organic carbon (TOC):TON ratio (1999–2017) per river (in dashed lines), and for all rivers combined (bold line), using loess smoother and showing standard errors (in grey per river, in red for overall trend). Load and load ratio data was log, and sqrt transformed, respectively, to ensure normal distribution of the response variables. The figure was generated using the freely available software R (R CoreTeam 2020, Version 4.0.2, https://www.R-project.org/).

Combining data from atmospheric and riverine monitoring programs in Norway, we found a significant declining trend in S deposition (− 5.04% annually, *p* < 0.001), and a weaker, but significant declining trend in N deposition since 1980 (− 0.42% annually, *p* < 0.001) (Table 1, Fig. 2a,b). In parallel, we found a decreasing trend in DIN loads (− 0.70% annually, *p* < 0.001) and an increasing trend in TON loads (2.11% annually, *p* < 0.001) in the five studied Norwegian rivers over the past 27 years (1990–2017) (Table1, 2) (Fig. 2c,d). Supporting hypothesis 1, these observed declines in DIN and increases in TON loads to the Skagerrak coast, were directly linked to the declining depositions in N and S, respectively (linear mixed effect model analysis, Table 2; Supplementary, Table S2–S6). Further, the recent changes in climatic variables (increasing temperature and precipitation), as well as increased discharge loads were important additional drivers of both the DIN decline and TON increase (Tables 1, 2). Lastly, also total N, as well as total P loads increased significantly over the studied time period (N: 0.45% annually, *p* < 0.001; P: 0.70% , *p* < 0.001), while DIN:TON, DIN:TP and TN:TP molar ratios decreased by 2.59, 1.84, 0.29% annually (all *p* < 0.001) (Table 1). These findings support hypothesis 2, illustrating that nutrient balances of river waters feeding into coastal zones have been shifting systematically in response to the declining anthropogenic S, and N deposition in Europe over the past decades. Supporting hypothesis 3, we found that while TOC loads in Norwegian rivers have been significantly increasing over the past 27 years (0.92% annually, *p* < 0.001), the TOC:TON ratio has been declining (− 1.09% annually, *p* < 0.001) (Fig. 2h). This finding indicates that more organically bound N (i.e. TON) has been processed and leached from boreal soils than carbon in the studied areas. However, further empirical studies are needed to determine the processes involved in the cycling and flux of the deposited N on the river catchments^27–29^, but also how these processes may be altered by multiple stressor interactions^26,30,31^. In sum, all observed trends could be correlated to human pressures on air (deposition), but especially also to the climate (precipitation, temperature) (Table 2; Supplementary, Table S2–S6).

**Table 1:** Significant trends (annual change (absolute), annual change %, and *p* value) of response variables in the investigated rivers over the study periods (time frames) using seasonal-regional Mann–Kendall trend test.

Variable	Time frame	Theil–Sen’s slope	Change % annual	*p*
S deposition (mg m^−2^ yr^−1^)	1980–2014*	− 12.06	− 5.04	< 0.001
DIN:TON (mol:mol)	1990–2017	− 0.04	− 2.59	< 0.001
DIN:TP (mol:mol)	1990–2017	− 1.38	− 1.84	< 0.001
TOC:TON (mol:mol)	1999–2017	− 0.28	− 1.09	< 0.001
DIN (µg m^−2^ yr^−1^)	1990–2017	− 93.94	− 0.70	< 0.001
N deposition (mg m^−2^ yr^−1^)	1980–2014*	− 2.18	− 0.42	< 0.001
TN:TP (mol:mol)	1990–2017	− 0.38	− 0.29	0.008
Precipitation (mm yr^−1^)	1971–2017	0.33	0.41	< 0.001
TN (µg m^−2^ yr^−1^)	1990–2017	98.07	0.45	< 0.001
Discharge (mm yr^−1^)	1990–2017	0.38	0.65	< 0.001
TP (µg m^−2^ yr^−1^)	1990–2017	2.40	0.70	< 0.001
TOC (mg m^−2^ yr^−1^)	1999–2017	1.75	0.92	< 0.001
TON (µg m^−2^ yr^−1^)	1990–2017	173.9	2.11	< 0.001
Air temperature (°C)	1971–2017	0.04	4.60	< 0.001

Yearly data with monthly resolution (January to December) was included in the analysis, except for the *Deposition parameters, where data from the following time periods was included: 1980, 1994, 1999, 2004, 2009, 2014.

**Table 2:** Drivers of significant trends in response variables identified via linear mixed effect modelling in the years 2004, 2009, 2014 (monthly resolution, January to December).

Variable	Stats	Interc	N_dep_	S_dep_	Year	Temp	Precip	Discharge	df N	R^2^
DIN^log^	Slope	43.789	0.002		− 0.022	− 0.031	− 0.001	0.015	115	0.92
	*p*	< *0.001*	*0.001*		< *0.001*	< *0.001*	*0.045*	< *0.001*	180	
TON^log^	Slope	1.624		− 0.001		0.009		0.013	117	0.74
	*p*	< *0.001*		*0.003*		*0.010*		< *0.001*	180	
DIN:TP^sqrt^	Slope	162.356	0.015		− 0.080	− 0.122	− 0.006		116	0.70
	*p*	*0.008*	< *0.001*		*0.009*	< *0.001*	*0.028*		180	
DIN:TON^sqrt^	Slope	60.827		− 0.001	− 0.030	− 0.023	0.000	0.000	115	0.63
	*p*	< *0.001*		< *0.001*	< *0.001*	< *0.001*	*0.016*	*0.803*	180	
TOC:TON^sqrt^	Slope	71.271			− 0.033	− 0.018		0.004	117	0.20
	*p*	< *0.001*			< *0.001*	< *0.001*		*0.001*	180	

Statistical results (Stats) are presented as slope, *p* value, and R^2^. For abbreviations: Interc. = Intercept, N_dep_ = N deposition, S_dep_ = S deposition, Temp = Temperature, Precip = Precipitation. Transformation of response variables (log- or square-root) to ensure normality is indicated by superscripts, “log” and “sqrt”, respectively. For detailed information on respective models see Supplementary Information (Table S2–S6).

Air pollution poses a threat to human health, but also the natural environment globally, e.g. by causing acidification and eutrophication of freshwaters^32^. To reduce and control air pollution and its negative impacts on aquatic systems, international agreements setting national emission targets for pollutants such as S and N have been in action for decades (e.g. 1979: UNECE Convention on Long-Range Transboundary Air Pollution). By illustrating the steady declines in both S and N deposition since the 1980s, our results confirm the success of such international agreements (Fig. 2a,b).

Despite the comparably small decline in N deposition, overall DIN loads, as well as DIN:TP ratios in the studied Norwegian rivers have declined significantly over the past three decades (Fig. 2c,e). This showcases that N emission control is an effective measure for reducing riverine DIN loads and that despite most of the deposited N being taken up by the terrestrial system, reductions in N deposition result in reduced leaching of N from catchments. Our results stand in line with a study from Sweden, showing that lake DIN concentrations, as well as DIN:TP ratios have declined across the country in response to declining N deposition from central Europe^4^. In their study, the authors could further prove that these changes in nutrient availability had strong effects on primary producers as declining N deposition resulted in a shift from N and P colimitation, towards N limitation alone. It is known that shifts towards N limitation may in many cases promote N-fixing cyanobacteria blooms^33^, especially when N limitation occurs synchronously with temperature increases^34^. Cyanobacteria may affect higher trophic levels due to their reduced food quality as well as toxic effects^35^. It is currently unknown whether declining DIN:TP ratios may systematically promote cyanobacteria blooms in Scandinavian freshwaters or downstream coastal systems, especially since the influence of multiple stressors on determining bloom situations may further complicate the forecasting of such shifts^36^. However, trends in increasing lake cyanobacteria blooms in southern Sweden have already been observed, indicating potential threats for water quality and lake ecosystem production^37^.

Synchronous to the DIN and DIN:TP declines, we found significant increases in riverine TON loads to Norwegian coastal ecosystems (Fig. 2d). This finding indicates a strong shift in the N nutrient stoichiometry from inorganic to organic fractions (Fig. 2f). Such a systematic shift has previously not been observed in Scandinavian rivers, but is not surprising given the observed freshwater browning and increased runoff of terrestrial organic material from boreal catchments^7,13,14^ and the simultaneous declines in DIN runoff due to successful N emission reductions^4–6^. Chemical parameters and especially nutrient stoichiometry may be excellent early warning indicators for net ecosystem changes in aquatic systems^38–40^. However, future research and monitoring programs need to move beyond using simple total nutrient contents, as well as beyond focusing dominantly on carbon compounds for assessing net ecosystem impacts^16–18,20^. Despite their ecological importance from fresh to salty habitats, nutrients (N and P) bound to organic material have to date caught comparably less attention^41–44^. Focusing on these compounds may be especially important for boreal freshwaters and coastal systems, where overall organic compounds (C, N, P) are systematically increasing, and additionally more bioavailable than previously thought^13,21,43–46^. If only measuring total N, P and TN:TP ratios as indicators of the ecosystem’s trophic state, as well as total C or dissolved organic C (DOC) as indicators for the organic matter runoff or browning, our study would not have succeeded in detecting any of the observed strong trends in riverine loads to the coast.

The TOC:TON ratio showed a decreasing trend over the studied time frame (Fig. 2h, Table 1), despite TOC loads steadily increasing especially since 2005 (Table 1; Supplementary, Fig. S1). This trend indicates that soluble organic matter is being enriched in organically bound N. This, coupled with the observed increasing trend in both TOC and TON deserves increased investigation^22,25^. As no strong leaching of DIN has been observed after N deposition to boreal catchments^47–49^, policy makers in Norway are currently debating whether to use N fertilization to improve the CO_2_ capture potential in boreal forests^50^. However, our study showcases that interactions between air (i.e. deposition), climate, vegetation, soil and water, as well as ongoing climatic changes (i.e. increasing precipitation, temperature, discharge) are complex, and net effects of N fertilization and processing within the catchment may be diverse. As examples, potential consequences of forest harvest may include increased TON leaching, but also changes in ground vegetation composition and the removal of base cations with effects on e.g. soil hydrology^47–49^. In Sweden and Finland, the N fertilization of forests is already common practice in order to increase forest biomass harvest^49^. However, if part of the added N may leach from boreal soils in organic form into freshwaters and potentially costal zones, this consequence and the involved processes deserve increased attention and may call for rethinking of N fertilization practices and policy.

Lastly, seasonality, regionality and climate may strongly impact timing, magnitude, processing, and quality of terrestrial material being flushed into downstream freshwater and coastal systems^22–24^. These links are also indicated by our results where all response parameters were strongly correlated to catchment temperature, precipitation and discharge (Table 2). For example, seasonality may play an important role explaining the coupling between riverine and downstream coastal nutrient concentrations, especially due to differences in water residence times^51–53^. Additionally, changes in vegetation cover and land use may affect the flux of nutrients through the catchment by affecting production, processing, as well as export of terrestrial material^7,29,54^. For our five studied catchments draining into the Skagerrak, no temporal data of vegetation cover, nor land use was available for the investigated timespan. Specific potential effects of altered vegetation cover, tree line advance and land use for the overall Skagerrak catchment remains to be tested in future studies.

In the Norwegian Skagerrak coast, as well as the North Sea, there has been an observed change in water clarity over the past decades, a phenomenon referred to as coastal darkening^10,11^. It is hypothesized that this phenomenon might be related to the increased riverine discharge of freshwater, as well as increased discharge of terrestrial organic matter into coastal zones^3,12^. The results of this study support this hypothesis, with increasing trends in both TOC and TON loads. However, future research needs to investigate whether long-term changes in coastal inorganic: organic ratios correspond to the large-scale shifts found in this study.

## Conclusion

Large changes have occurred in Norwegian rivers over the past decades regarding N loads and stoichiometry, where the organic nitrogen fraction has been steadily increasing. This study showcases (1) how closely air, land, and water systems are connected, (2) that national and international policies on non-aquatic compartments such as air, may have a strong net influence on aquatic ecosystems, (3) that chemical parameters and nutrient ratios may be excellent early warning indicators for net ecosystem changes, and lastly, (4) how important long-term data sets are to test for water quality changes in response to multiple stressors.

## Methods

### Data sources

We analysed time series data for five major Norwegian rivers and associated catchment data to assess trends in, and potential drivers of nutrient loads and nutrient load ratios (i.e. stoichiometric ratios) (Fig. 1; Supplementary, Table S1). The river data stems from the Norwegian river monitoring program^55^, including discharge data from the Norwegian Water Resources and Energy Directorate (NVE) and concentration data collected by the Norwegian Institute for Water Research. The supporting data includes deposition data from the Norwegian monitoring program on long-range transboundary air pollution in Norway collected by the Norwegian Institute for Air Research^56^, temperature and precipitation data from the Norwegian Meteorological Institute^57,58^ and land use data extracted from the public mapping service website of NVE (nevina.nve.no). The trend analyses was run for the complete time periods where continuous data was available, i.e. from 1971 for temperature and precipitation, from 1980 for S and N deposition, from 1990–2017 for discharge, as well as all nutrient related parameters except total organic carbon (TOC) and TOC related parameters. Here, data was available from 1999 as TOC was not added to the monitoring program before that time point for several of the rivers. Mixed effect model analysis, to investigate the drivers behind the respective trends, was conducted for selected time periods where data was available for all relevant parameters with monthly resolution to ensure a balanced statistical design (i.e. selected years were: 2004, 2009, 2014, sample size = 180).

### River data

The rivers selected for this study are the five major rivers in the south eastern part of Norway, and all drain to the Skagerrak coast (Fig. 1). Part of these catchments are among areas most heavily affected by atmospheric deposition in Norway, with a gradient decreasing from south to north (Fig. 2a,b)^56^. Although within the same region, the catchments show a range in catchment size and land use (Fig. 1; Supplementary, Table S1). Water sampling was conducted close to the river mouth, to be representative for calculating the total load from the catchment. The samples were collected monthly, following standard procedures^55^. The parameters selected for this study were total N (TN), total P (TP), total organic C (TOC), dissolved inorganic N (DIN = NO_3 _+ NH_4_), and total organic N (TON = TN − DIN). Most of these parameters have been analysed since the start of the program in 1990, but NH_4_ was not included until 1992. However, given that the NH_4_ contribution to DIN and TN was comparably small (9 and 5%, respectively), the analysis was conducted for the whole time period (i.e. DIN in 1990 and 1991 only represents NO_3_ loads). Thus, any detected trends in DIN or TON loads and load ratios may be considered conservative in terms of the hypotheses, given that the initial DIN and TON loads are slightly lower and higher, respectively, than the actual loads. TOC was not measured in all rivers until 1999, wherefore analysis of TOC-related parameters was restricted to this shorter time period (i.e. data included 1999–2017) (Supplementary, Fig. S1). For load calculations, concentration data was linearly interpolated to daily values and then multiplied by daily discharge to give daily loads, which were subsequently summed to monthly values.

Nutrient ratios were calculated as molar ratios, based on the summarised load values; i.e. for the load ratios to be calculated from comparable loads, samples where certain parameters were missing with respect to the parameters that were generally analysed for the specific year/river were excluded. The ratios DIN:TON, TN:TP, DIN:TP, and TOC:TON were selected for the analysis. The N:P elemental ratios can be used as indicators for biological nutrient limitation. Especially the DIN:TP ratio has been shown to be a robust predictor of e.g. phytoplankton nutrient limitation in boreal lakes and coastal regions^18,19^ and in other areas with high dissolved organic matter^59^ compared to TN:TP.

### Meteorological and deposition data

Daily air temperature and precipitation was available on a 1 km × 1 km grid covering the whole of Norway. Gridded data was averaged for each catchment using the intersection between the delineated catchment and the grid cells, weighting according to grid areas. Monthly values were calculated by summing or averaging across daily values for precipitation and temperature, respectively. Data for 1990 to 2017 was used in the analysis of meteorological data.

Atmospheric deposition data was provided as five-year average total inorganic N and sea-salt corrected S deposition (wet + dry), on a 0.25° × 0.125° grid^56^. As for meteorological data, a weighted average deposition per catchment was calculated based on the intersection between the catchment and the deposition grid cells. Data from the following time periods was used: 1978–1982, 1992–1996, 1997–2001, 2002–2006, 2007–2011, and 2012–2016 (1978–1982 only included in figure, i.e. Fig. 2a,b). The middle year for each time range was used in the data analysis (e.g. 2014 for time period 2012–2016).

### Data analysis and statistical methods

We analysed N loads, load ratios, discharge as well as meteorological data (N, S deposition, temperature, precipitation) for monotonic time series trends. Specifically, we conducted non-parametric seasonal (i.e. month)-regional (i.e. river) Mann–Kendall (MK) test using the “rkt” package in R^60^. The seasonal-regional MK test is an extension of the MK test, accounting for systematic, unidirectional seasonality and regionality effects in the time series trends^60^. Specifically, a monotonic upward or downward trend means that the variable consistently increases or decreases (respectively) through time, without, however, implying a linear trend per se. Trends are presented as the overall Theil–Sen’s slope, as well as the calculated percentage change in the mean quantity (e.g. load quantity) per year (Table 1).

To identify and describe the drivers behind the observed significant time trends in DIN and TON loads, as well as DIN:TON, DIN:TP, and TOC:TON molar ratios we conducted linear mixed effect modelling (LME) following standard protocols for model selection and validation^61,62^ and using the “nlme” package in R^63^. Prior to analysis, load and load ratio data were log, and square root transformed, respectively, to ensure normal distribution of the response variables. In step 1 of the LME analysis, the random structure of the model was selected based on prior knowledge of the dependency structure in the data (i.e. month nested in river, note that year was kept as a fixed effect as only three levels). In step 2, the model was fitted and the importance and significance of fixed effects evaluated using the likelihood ratio (ML) test to compare alternative models in a stepwise selection process by using the Bayesian information Criterion (AIC) to inform selection to gain the best explanatory model. Initial selected drivers were N or S deposition (N for DIN related parameters, S for TON related parameters), year, temperature, precipitation and discharge (Table 2). Note that no interaction terms were included to avoid overfitting. Lastly, models were validated based on investigating the normalised residuals (using restricted maximum likelihood estimation (REML)) to identify potential violation of homogeneity, as well as verify normality by using the sjPlot package^64^ in addition to base packages. For details on model results see Supplementary, Table S2–S6. The graphics of this study (Figs. 1, 2; Supplementary, Fig. S1) were produced using the R package “ggplot2”^63^. All statistical analyses, as well as graphics were conducted using the freely available software R (R CoreTeam 2020, Version 4.0.2, https://www.R-project.org/).

## Supplementary information

Supplementary information.

## Data Availability

Raw data were collected, and are administered by the Norwegian Water Resources and Energy Directorate (discharge data), the Norwegian Institute for Air Research (deposition data), the Norwegian Meteorological Institute (meteorological data), and the Norwegian Institute for Water Research (water related parameters). Derived data supporting the findings, as well as R code to reproduce figures and analysis of this study are available from the corresponding author upon request.
