# Supplementary material for: Organic nitrogen steadily increasing in Norwegian rivers draining to the Skagerrak coast
Source: Sci Rep. 2020 Oct 28;10:18451. doi: 10.1038/s41598-020-75532-5 (PMC7595164; doi:10.1038/s41598-020-75532-5)

Supplementary Information

Title: Organic nitrogen steadily increasing in Norwegian rivers draining to the Skagerrak coast

Authors: Deininger A., Kaste Ø., Frigstad H., and Austnes K.

Table with legend

Table S1. Background characteristics of the five Norwegian rivers included in the analysis (exact location of monitoring station, and catchment area).

River	UTM North (m)	UTM East (m)	Catchment area (km^2)
Drammenselva	6624446	556695	17034
Glomma	6573156	621600	41918
Numedalslågen	6551828	561347	5577
Otra	6450006	438495	3738
Skienselva	6562920	534741	10772

Table S2. Details of linear mixed effect model for parameter: dissolved inorganic nitrogen (load in mg m^{-2} yr^{-1}, DIN). Note log transformation to ensure normality of response variable. For abbreviations: CI = confidence intervals, p = p – value, Interc. = Intercept, Ndep = N deposition, Sdep = S deposition, Temp = Temperature, Precip = Precipitation, Dis = Discharge.

		**L.DIN**	
Predictors	*Estimates*	*CI*	*p*
(Intercept)	43.79	26.38 – 61.20	**<0.001**
Ndep	0.00	0.00 – 0.00	**0.001**
Precip	-0.00	-0.00 – -0.00	**0.045**
Temp	-0.03	-0.04 – -0.03	**<0.001**
Dis	0.01	0.01 – 0.02	**<0.001**
Year	-0.02	-0.03 – -0.01	**<0.001**
**Random Effects**			
σ^2	0.05		
$\tau_{00\ Month}$	0.81		
$\tau_{00\ River}$	0.06		
N_{Month}	12		
N_{River}	5		
Observations	180		
Marginal R^2	0.921		

Table S3. Details of linear mixed effect model for parameter: total organic nitrogen (load in mg m^{-2} yr^{-1}, TON). Note log transformation to ensure normality of response variable. For abbreviations: CI = confidence intervals, p = p – value, Interc. = Intercept, Ndep = N deposition, Sdep = S deposition, Temp = Temperature, Precip = Precipitation, Dis = Discharge.

	**L.TON**		
Predictors	*Estimates*	*CI*	*p*
(Intercept)	1.62	1.35 – 1.90	**<0.001**
Sdep	-0.00	-0.00 – -0.00	**0.003**
Temp	0.01	0.00 – 0.02	**0.010**
Dis	0.01	0.01 – 0.01	**<0.001**
**Random Effects**			
σ^2	0.06		
$\tau_{00\ Month}$	0.15		
$\tau_{00\ River}$	0.13		
N_{Month}	12		
N_{River}	5		
Observations	180		
Marginal R^2	0.744		

Table S4. Details of linear mixed effect model for parameter: dissolved inorganic nitrogen to total phosphorus (ratio in molar:molar, DIN:TP). Note square-root transformation to ensure normality of response variable. For abbreviations: CI = confidence intervals, p = p – value, Interc. = Intercept, Ndep = N deposition, Sdep = S deposition, Temp = Temperature, Precip = Precipitation, Dis = Discharge.

	**S.DIN.TP**		
Predictors	*Estimates*	*CI*	*p*
(Intercept)	162.36	43.06 – 281.66	**0.008**
Ndep	0.02	0.01 – 0.02	**<0.001**
Precip	-0.01	-0.01 – -0.00	**0.028**
Temp	-0.12	-0.16 – -0.08	**<0.001**
Year	-0.08	-0.14 – -0.02	**0.009**
**Random Effects**			
σ^2	2.66		
$\tau_{00\ Month}$	2.71		
$\tau_{00\ River}$	0.63		
N_{Month}	12		
N_{River}	5		
Observations	180		
Marginal R^2	0.704		

Table S5. Details of linear mixed effect model for parameter: dissolved inorganic nitrogen to total organic nitrogen (ratio in molar:molar, DIN:TON). Discharge (Dis) was kept in the model as likelihood ratio (ML) test to compare alternative models (with/without Dis as fixed term) showed its importance for final model (Zuur et al. 2009). Note square-root transformation to ensure normality of response variable. For abbreviations: CI = confidence intervals, p = p – value, Interc. = Intercept, Ndep = N deposition, Sdep = S deposition, Temp = Temperature, Precip = Precipitation, Dis = Discharge.

	S.DIN.TON		
Predictors	*Estimates*	*CI*	*p*
(Intercept)	60.83	42.43 – 79.22	**<0.001**
Sdep	-0.00	-0.00 – -0.00	**<0.001**
Precip	-0.00	-0.00 – -0.00	**0.016**
Temp	-0.02	-0.03 – -0.02	**<0.001**
Dis	0.00	-0.00 – 0.00	0.803
Year	-0.03	-0.04 – -0.02	**<0.001**
**Random Effects**			
σ^2	0.03		
$\tau_{00\ Month}$	0.00		
$\tau_{00\ River}$	0.07		
N_{Month}	12		
N_{River}	5		
Observations	180		
Marginal R^2	0.630		

Table S6. Details of linear mixed effect model for parameter: total organic carbon to total organic nitrogen (ratio in molar:molar, TOC:TON). Note square-root transformation to ensure normality of response variable. For abbreviations: CI = confidence intervals, p = p – value, Interc. = Intercept, Ndep = N deposition, Sdep = S deposition, Temp = Temperature, Precip = Precipitation, Dis = Discharge.

	**S.TOC.TON**		
Predictors	*Estimates*	*CI*	*p*
(Intercept)	71.27	38.93 – 103.61	**<0.001**
Temp	-0.02	-0.03 – -0.01	**<0.001**
Dis	0.00	0.00 – 0.01	**0.001**
Year	-0.03	-0.05 – -0.02	**<0.001**
**Random Effects**			
σ^2	0.18		
τ_{00} Month	0.08		
τ_{00} River	0.05		
N Month	12		
N River	5		
Observations	180		
Marginal R^2	0.203		

Figures with legends

Figure S1. Time series of total organic carbon (TOC) related parameters. Here data was only included from 1999 – 2017 due to lack of monitoring prior to 1999. The figure was generated using the freely available software R (R CoreTeam 2020, Version 4.0.2, https://www.R-project.org/).

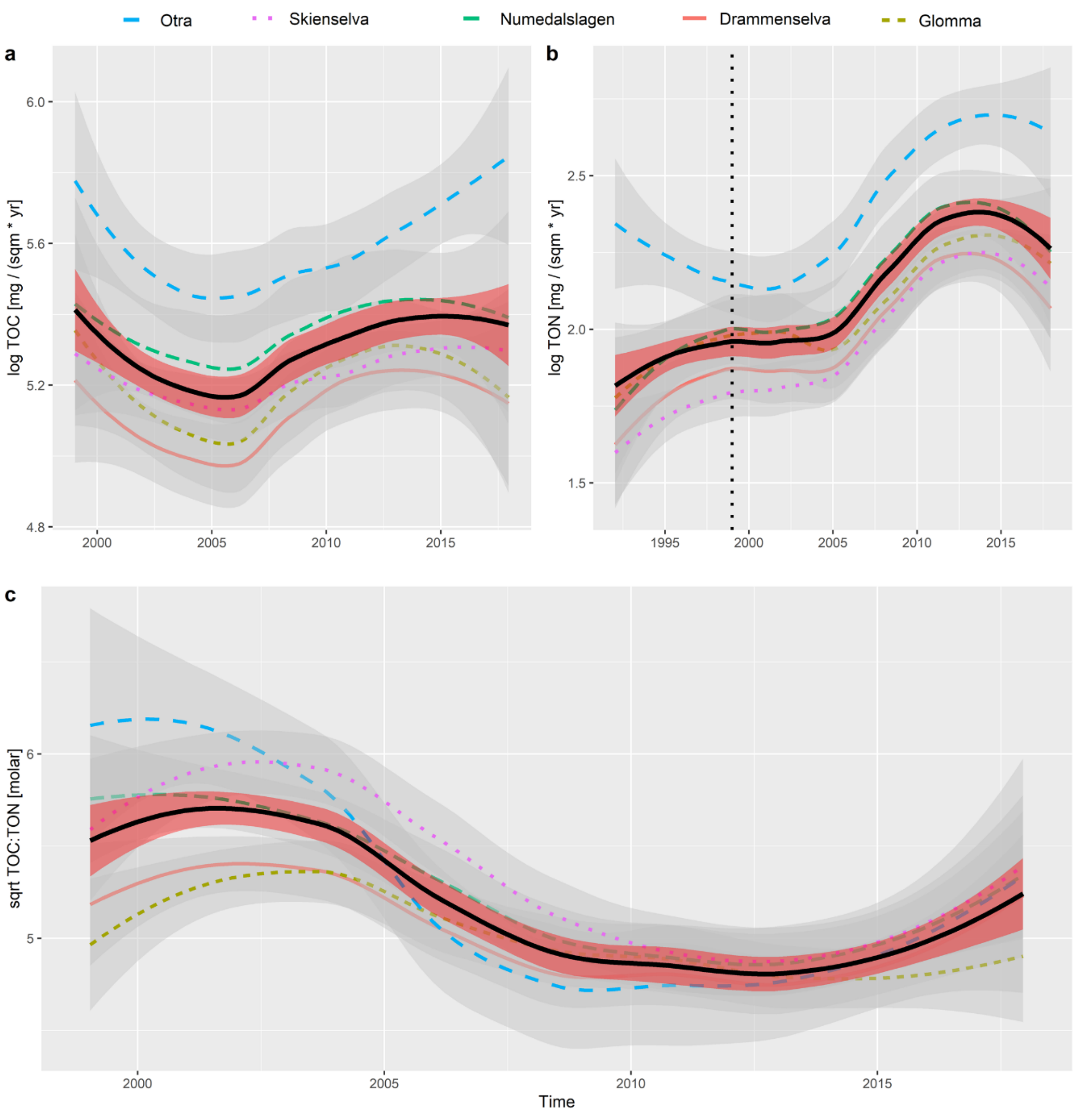

Supplement: Supplementary file 1 — Supplementary information. [file 41598_2020_75532_MOESM1_ESM.pdf]
